# The Impact of Household Dysfunction on Dating Violence Perpetration Among Adolescents in the United States: A Scoping Review

**DOI:** 10.1177/15248380241277267

**Published:** 2024-09-19

**Authors:** Sumaita Choudhury, Melissa F. Peskin, Timothy J. Walker, Emily T. Hébert, Nivedhitha Parthasarathy, Kaitlyn L. Zajack-Garcia, Lea Sacca, Christine M. Markham

**Affiliations:** 1 The University of Texas Health Science Center at Houston School of Public Health (UTHealth School of Public Health), USA; 2 The University of Texas Health Science Center, Austin (School of Public Health), USA; 3 Charles E. Schmidt College of Medicine, Florida Atlantic University, Boca Raton, USA

**Keywords:** adverse childhood experience, household dysfunction, dating violence, family conflict, adolescents

## Abstract

Adverse childhood experiences, such as household dysfunction (HD), play a central role in how adolescents establish, experience, and navigate the challenges of relationship formation, maintenance, and dissolution. HD exposures have been independently associated with dating violence (DV) perpetration in both adolescents and adults. However, research examining the association between the concurrent effect of HD on DV perpetration, especially among adolescents remains scarce. Thus, we conducted a scoping review to accumulate and summarize existing research regarding the impact of HD on DV perpetration among adolescents aged 10 to 17 years in the United States. We used three electronic databases, Medline (Ovid), PsycINFO, and EMBASE, to search for studies published in English between 2013 and August 2023. A total of 14 studies were retained for this review after full-text screening. Most of the included studies (64%) were longitudinal. Concerning HD measurement, 71% of studies evaluated witnessing intimate partner violence (IPV), and the remaining 29% assessed family conflict, both using different instruments. Regarding DV measurement, 43% of studies utilized the Safe Dates Abuse measures to assess various forms of DV perpetration. Findings from 3/4 (75%) studies that evaluated family conflict found it to be a significant predictor of DV perpetration. Additionally, 8/10 (80%) studies that assessed exposure to IPV reported significant associations with various forms of DV perpetration among adolescents. None of the included studies measured HD comprehensively; thus, measurement development is imperative. Findings from this review may help initiate the development of a more comprehensive HD measure, promote early intervention, and foster resilience among adolescents.

## Introduction

Adolescent dating violence (DV) is a serious public health concern in the United States (CDC, 2022; Peskin et al., 2019; Reidy et al., 2015; Vagi et al., 2013). Adolescent DV is a type of intimate partner violence (IPV) that includes (a) physical DV, (b) sexual DV, (c) psychological/emotional DV (i.e., threatening, verbal, relational, and digital/cyber), and (d) stalking (CDC, 2022; Shorey et al., 2019). The prevalence of DV victimization is likely underestimated among adolescents in the United States due to underlying factors such as social stigma, fear, and measurement-related limitations regarding how DV questions are assessed across national surveys (Basile et al., 2006; Peskin et al., 2019; Rothman & Xuan, 2014). However, the 2021 CDC Youth Risk Behavior Surveillance System survey reported that among high school adolescents who were in a relationship in the past year, nearly 9.7% reported sexual DV and 8.5% reported physical DV (CDC, 2023; Clayton, 2023). Further, DV perpetration rates are scant compared to victimization rates, and existing literature tends to concentrate on victimization rather than comprehensively exploring risk factors for perpetration, including adverse childhood experiences (ACEs) among adolescents (Boyce et al., 2023).

ACEs, such as childhood maltreatment, household dysfunction (HD), and community violence, have been associated with DV victimization and perpetration both in adolescence and adulthood; however, there is limited empirical research examining the relation between the HD domain and DV perpetration, especially among adolescents (Afifi et al., 2020; Duke et al., 2010; Haynie et al., 2013; Jouriles et al., 2012; Malhi et al., 2020; Navarro et al., 2022; Wolfe et al., 2005). HD is one of the domains of ACE that has been described as children growing up in an environment that threatens a child’s stability and welfare due to witnessing parental IPV (Afifi et al., 2020). Due to HD’s multifaceted and subjective nature, there is no set definition of the HD construct. However, according to the original ACE study, HD encompasses witnessing parental IPV, parental divorce/separation, living with household member(s) with substance use and mental health disorders, and family history of incarceration (Felitti et al., 2019). In addition, recent studies have expanded upon the definition and categories of ACE. They identified additional forms of HD, including family chaos, parental harsh discipline, family violence, death in the family, and living with household member(s) with disabilities (Afifi et al., 2020; Bussemakers et al., 2022; Davis et al., 2019; Felitti et al., 2019; Gottfredson et al., 2022; Morris et al., 2015). It is important to note that most of the existing ACE measures only include some of these additional forms of HD and usually assess them as individual constructs rather than assessing HD’s overall impact. Since romantic or dating relationships usually begin during middle school, which is a critical period of time when adolescents develop their cognitive abilities, interpersonal relationships, independence, social connectedness, and communication skills, adverse experiences such as HD can further hinder adolescents from initiating and maintaining their healthy dating relationships (Collins, 2003; Malhi et al., 2020; Simon et al., 2008).

Further research is warranted regarding the association between experiencing HD, specifically, and increased risk for DV perpetration among adolescents. For example, a study that explored the association between various forms of ACEs and adolescent DV perpetration indicated that approximately 28.9% of adolescents reported at least one ACE, primarily substance use disorder by a household member, which was significantly correlated with adolescents’ DV perpetration, bullying, physical fights, and other maladaptive behaviors (Duke et al., 2010). In addition, research has shown that adolescents living in a dysfunctional environment or suffering from any negative experiences are at heightened risk for extreme emotional distress resulting in relationship problems, breakups, a decline in cognitive skills, challenges with self-esteem and autonomy, and other psychological problems (Malhi et al., 2020; Navarro et al., 2022; Peskin et al., 2019; Wincentak et al., 2017).

Previous scoping reviews have supported the link between ACEs (including HD) and DV. For example, one scoping review that investigated DV perpetration by male adolescents and young adults aged 10 to 24 years within an international context found that individuals who experienced ACEs were more likely to perpetrate violence in relationships (Malhi et al., 2020). However, that scoping review was limited because it did not assess the effect of HD specifically on both male and female DV perpetration, particularly among adolescents in the United States (Malhi et al., 2020). Another scoping review examined the association between ACEs within the family context (i.e., witnessing IPV, parental neglect, etc.) and physical and online DV among adults. Yet, this review reported direct associations only between particular types of ACEs and DV victimization and perpetration (Navarro et al., 2022). Navarro et al. (2022) also found that emotional, sexual, and physical abuse, witnessing IPV, parental mental illness, HD, and poor relationships with parents were strongly associated with DV perpetration among adults. However, this review did not address the gap in evaluating the relation between HD and any type of DV perpetration (i.e., physical, sexual, psychological, online, etc.) simultaneously among adolescents in the United States.

Existing studies that identified ACEs as a predictor of DV perpetration have predominantly examined ACEs’ impact on IPV in adulthood rather than assessing the concurrent impact of HD on adolescents’ dating perpetration behaviors (Duke et al., 2010; Fonseka et al., 2015). Moreover, no comprehensive reviews have been undertaken to investigate the impact of HD on various types of DV perpetration, especially among adolescents in the United States. Thus, the primary goal of this scoping review is to summarize results from existing research studies on how HD impacts DV perpetration behaviors among adolescents ages 10 to 17 years in the United States, address research gaps and limitations, and discuss the next steps for future research.

## Methods

This scoping review followed the five-step methodological framework by Arksey and O’Malley (2005). The five steps of the framework include (a) identification of the research question; (b) identifying relevant studies; (c) selection of studies; (d) charting the data; and (e) collating, summarizing, and reporting the results (Arksey & O’Malley, 2005). The Preferred Reporting Items for Systematic Reviews and Meta-Analyses extension for Scoping Reviews (PRISMA-ScR) checklist was also used as a reference for study sections to follow the guidelines for conducting and publishing scoping reviews.

### Protocol and Registration

The scoping review protocol was registered under the Open Science Framework (OSF) (https://osf.io/gpcru/) on December 12, 2022, before starting the search strategy process for relevant research studies for this review. An updated protocol version was uploaded to OSF on August 22, 2023.

### Step 1: Identification of the Research Question

The research question guiding this scoping review is: “What is the impact of HD on DV perpetration among adolescents aged 10 to 17 years in the United States?”

### Step 2: Identifying Relevant Studies

This scoping review used these three electronic databases for the literature search since they encompass articles relevant to our research topic: Medline (Ovid), EMBASE, and PsycINFO. The search strategy was developed with the support of a research librarian at the University of Texas Health Science Center in Houston. The literature search strategy includes subject headings, keywords, and phrases on each concept of the research objective of this review (i.e., HD, ACEs, adolescents, and DV perpetration), which was translated to each database using Boolean operators (“OR” and “AND”) to combine, narrow, or widen the literature searches pertaining to the concepts of this scoping review research objective. The search strategies for each database are presented in the appendix section (Supplemental Material Appendix A: Search Strategy of all Databases). SC conducted the literature search on August 16, 2023, and the articles from the three databases were uploaded to the Rayyan platform for the initial study screening process. The duplicate articles were then removed using the Rayyan article duplicate detection tool (*Rayyan*, n.d.). After that, review members were added to Rayyan to begin the screening process (*Rayyan*, n.d.).

### Step 3: Selection of Studies

Authors SC, NP, and KZG independently reviewed and screened the titles and abstracts of the articles, following the eligibility criteria for this scoping review. After completing each database’s study title and abstract screening process, SC extracted the relevant studies and uploaded them into Rayyan separately for full-text review (*Rayyan*, n.d.). After that, SC, NP, and KZG conducted a full-text review of the selected studies based on this review’s eligibility criteria from September 2023 to October 2023. Finally, the senior author (CM) resolved any differences between the reviewers regarding the articles both during the titles/abstracts and full-text screening process.

### Eligibility Criteria

We used the PECO (Population, Exposure, Comparator, and Outcome) framework to develop the study eligibility criteria. The eligibility criteria for this scoping review are as follows: (a) adolescents aged between 10 and 17 years of age; (b) quantitative observational studies (i.e., cross-sectional, cohort, case-control, longitudinal, and trials (which includes baseline data)); (c) studies investigating at least one type of HD as an exposure (i.e., witnessing IPV/domestic violence/household violence, family violence, family chaos, family conflict, harsh parenting, parental conflict, parental aggression, parental divorce, separation, absence, parental death/sibling death/household member death, household member substance use disorder, household member mental health illness, household member incarceration, jail, criminal history, and household member with physical illness or disability); (d) at least one type of adolescent DV perpetration has to be included as one of the outcomes in the studies (i.e., physical DV, sexual DV, psychological or emotional DV, threatening DV, digital or cyber DV, and stalking); (e) published in English in a peer-reviewed journal; (f) published between 2013 and August 2023; and (g) conducted in the United States. The detailed study eligibility criteria are presented in Table 1.

**Table 1.** Scoping Review Study Eligibility Criteria.

PECO Framework	Inclusion	Exclusion
Population	Adolescents aged between 10 and 17 years.	The mean or median age of children/adolescents is not within the inclusion age range.
Exposure (IV)	Any form of household dysfunction (HD): • Witnessing intimate partner violence/domestic violence/household violence. • Family violence, family conflict, harsh parenting, parental conflict, parental aggression. • Parental divorce, separation, absence. • Parental death/sibling death/household member death. • Household member substance use disorder. • Household member mental health illness. • Household member incarceration, jail, criminal history. • Household member with physical illness or disability.	Only includes all other Adverse childhood experiences (ACEs): • Sexual or physical abuse/neglect. • Neighborhood or community violence. • Peer victimization. • Bullying • Natural disasters. • War-related trauma. • ACEs are reported as an overall composite score where HD is included but not directly measured in association with the outcome. • The HD category is measured as a moderator or a mediator and not measured as a single exposure or an independent variable regarding dating violence (DV) perpetration (outcome).
Comparator	N/A	N/A
Outcome (DV)	Any form of DV perpetration or aggression. • Physical DV • Sexual DV • Psychological or Emotional DV • Threatening DV • Digital or Cyber DV • Stalking	Only includes: • DV victimization • DV perpetration and victimization are combined into a composite score. • Bullying • Violence that is not related to dating or romantic relationships.
Study design	Quantitative studies • Cross-sectional • Cohort • Case-control • Longitudinal, and • Trials (which includes baseline data)	• Reviews • Qualitative studies • Research letters • Editorials • Commentaries • Reports • Dissertations, Conference • Proceedings/abstracts. • Trials that do not include findings from baseline data.
Limits	• Location: United States. • Published: 2013–2023 (August 16)—when the final search was conducted. • Language: English	• Other countries besides the United States • Published before 2013 • Languages other than English

### Steps 4 and 5: Charting the Data and Collating, Summarizing, and Reporting the Results

The Garrard Matrix method was used to extract, chart, and synthesize the findings from the included studies (Garrard, 2020). The matrix consists of study number, study author(s)/publication year, study aim(s), study design, sample size, type(s) of HD exposure measured in the studies (i.e., witnessing domestic violence, divorce, household member substance use disorder, etc.) along with the instrument(s) used, type(s) of DV perpetration measured as an outcome(s) in the studies (i.e., physical, sexual, psychological, stalking, threatening, verbal/emotional, relational, and digital) and the instrument(s) used, and the key findings of the studies. This information was charted in an Excel spreadsheet SC developed and reviewed by all authors.

The lead author, SC, evaluated the rigor and quality of the study using the Critical Appraisal Skills Programme (CASP) checklist (Center for Evidence-Based Management, 2014). This checklist has been utilized by previous scoping reviews (Navarro et al., 2022; M. E. Reyes et al., 2021). The CASP checklist implemented in this scoping review encompasses the following criteria: (a) clarity of stated study aims and objectives; (b) appropriateness of study methods; (c) adequate description of the methodology; (d) was there any bias in sample selection; (e) representativeness of the sample for generalizability of study results; (f) utilization of statistical power analysis for sample size calculation; (g) response rate; (h) utilization of reliable and valid measures; (i) examination for statistical significance; and (j) inclusion of confidence intervals (CI) in study findings (Center for Evidence-Based Management, 2014). The response to each criterion is classified as “yes,” “no,” or “unsure,” and a quality score for each study was derived by summing the number of “yes” responses. However, for item 4, the reverse score “no” was counted within the study rigor indices that contributed to the overall quality score of each included study (Center for Evidence-Based Management, 2014; M. E. Reyes et al., 2021). The CASP checklist findings are presented in Table 2.

**Table 2.** Critical Appraisal Skills Programme (CASP) Checklist for Assessing Study Rigor and Quality.

Study #	Study Aim(s)	Study Design	Selection of Subjects	Selection Bias^a^	Sample Generalizability	Stat Power	Response Rate	Valid Measures	Stat Sig	CI	Quality Score
1	Yes	Yes	Yes	Unsure	No	No	No	Yes	Yes	Yes	6
2	Yes	Yes	Yes	Unsure	Unsure	No	No	Yes	Yes	Yes	6
3	Yes	Yes	Yes	Unsure	No	No	No	Yes	Yes	Yes	6
4	Yes	Yes	Yes	Unsure	Unsure	No	Yes	Yes	Yes	Yes	7
5	Yes	Yes	Yes	Unsure	No	No	No	Yes	Yes	No	5
6	Yes	Yes	Yes	Unsure	No	No	No	Yes	Yes	Yes	6
7	Yes	Yes	Yes	Unsure	No	No	No	Yes	Yes	Yes	6
8	Yes	Yes	Yes	Yes	No	No	No	Yes	Yes	No	5
9	Yes	Yes	Yes	Unsure	No	No	No	Yes	Yes	No	5
10	Yes	Yes	Yes	Yes	No	No	No	Yes	Yes	Yes	6
11	Yes	Yes	Yes	Unsure	Yes	No	No	Yes	Yes	Yes	7
12	Yes	Yes	Yes	Unsure	No	No	No	Yes	Yes	Yes	6
13	Yes	Yes	Yes	Unsure	Unsure	No	No	Yes	Yes	No	5
14	Yes	Yes	Yes	Unsure	No	No	No	Yes	Yes	Yes	6

*Note.* CI = confidence intervals; quality score = total number of indicators reported by each study, which is marked as yes; Sig = significance; Stat = statistical.

a Indicator scored in a reversed manner.

## Results

**Figure 1.**
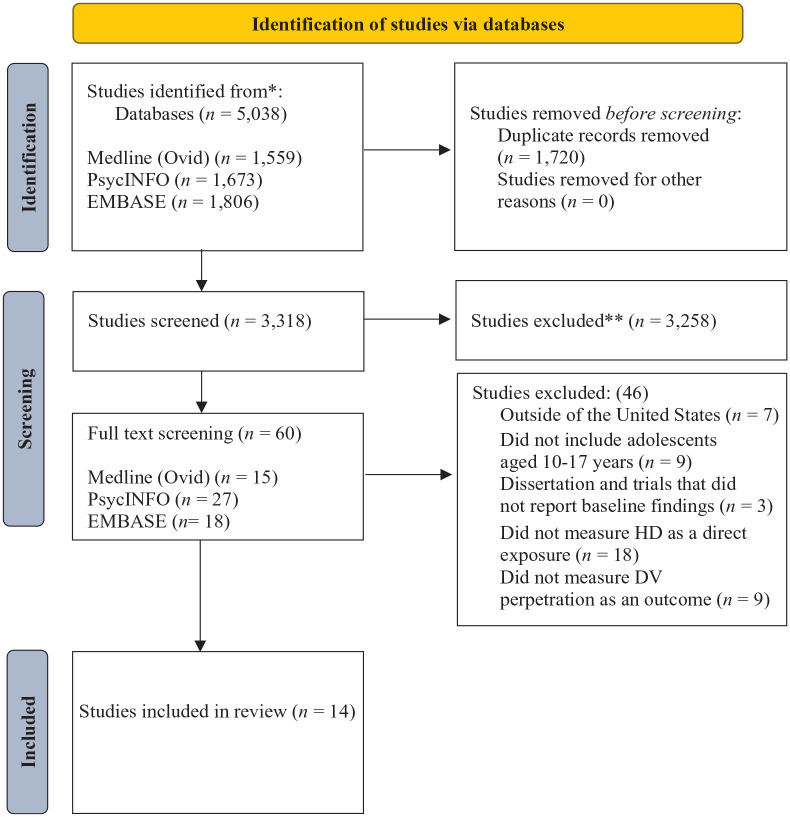
Preferred Reporting Items for Systematic Reviews and Meta-Analyses flow chart.

The literature search yielded a total of 5,038 studies (Figure 1 PRISMA diagram). Consequently, 1,720 studies were excluded due to duplication, and 3,318 studies were extracted for title and abstract screening. A total of 3,258 studies were excluded due to not meeting this review’s eligibility criteria, and 60 studies were retained for full-text screening. During the full-text review phase, 46 studies were excluded due to ineligibility, and 14 eligible studies were retained for analysis for this scoping review.

### Characteristics of Included Studies

The summaries of the included studies are presented in (Supplemental Material Appendix B: Garrard Matrix of Included Articles (*N* = 14)). Among the 14 included studies, *n* = 9 (64%) utilized a longitudinal study design, and *n* = 5 (36%) were cross-sectional. In addition, *n* = 6 (43%) had a sample size between 1,307 and 4,277 adolescents, and *n* = 5 (36%) included a sample size between 409 and 917 adolescents. One included study reported the smallest sample size of 139 adolescents; in contrast, the two remaining studies that assessed the impact of a DV prevention intervention reported the largest sample size of 15,863 adolescents during baseline data collection. Most studies *n* = 12 (86%) included male and female adolescents in their study population; of the remaining two studies, one only included male adolescents, and the other recruited only female adolescents.

Regarding the age range of adolescents in the included studies, 50% reported the mean age range of their study population between 11.8 and 16.3 years; however, the other 50% did not report a mean age of their study sample but did mention that adolescents who participated in their research were middle and/or high school students during study implementation. Finally, 36% of studies were conducted in North Carolina, 21% in Texas, and 14% in Kentucky, while one study recruited their study sample from various states (i.e., Chicago, Florida, Maryland, and California), and the remaining three studies did not mention a specific geographic location.

### Measurement of HD

The measures that were used to assess HD among the included studies varied greatly even though the studies examined only three types of HD, encompassing family conflict among household members (i.e., getting angry or yelling bad things at each other), witnessing IPV among parents (i.e., witnessing hitting, slapping, punching, pushing, choking, or threatening between their parents or parent’s dating partner), and harsh discipline (i.e., adolescents experiencing yelling, slapping or hitting from their parents). Four (29%) of the retained studies assessed *family conflict* using three different reliable measures, including the Family Conflict and Hostility Scale (Thornberry et al., 2003), Family Conflict Scale (Simpson & McBride, 1992), and Family Conflict Scale (Bloom, 1985). It should be noted that even though three different instruments were used to assess family conflict across these four studies, all of them examined the same types of family conflict scenarios using three measure items (i.e., how often or within the last 3 months that there were situations where family members were yelling bad things to each other, getting angry, or fighting with one another) (Davis et al., 2019; Foshee et al., 2015, 2016; Gottfredson et al., 2022).

The ten (71%) remaining studies examined *witnessing IPV/interparental violence between parents*, and one of these ten studies also assessed *parental harsh discipline*. Similar to the four studies that assessed family conflict using different measures, these ten studies used various measures to assess the same construct, which is witnessing IPV/interparental violence. For example, one study utilized two items from the Juvenile Victimization Questionnaire (Hamby et al., 2011) to examine whether adolescents witnessed or heard IPV between their parents, such as yelling or hitting (Latzman et al., 2015). Another study used four items from the Family Background Questionnaire (McGee et al., 1997) to measure adolescents’ exposure to IPV between their parents or caregivers (Moretti et al., 2014).

Out of the ten studies, four studies developed a one-item measure, which assessed the number of times adolescents had witnessed parental physical conflict (A. Mennicke et al., 2021; A. M. Mennicke et al., 2022; Reyes & Foshee, 2013; Reyes et al., 2015). Moreover, two of these ten studies applied a two-item measure developed by the authors that measured adolescents witnessing interparental violence related to pushing, shoving, or choking behaviors between parents (Temple, Shorey, Fite, et al., 2013; Temple, Shorey, Tortolero, et al., 2013). Similarly, one of the studies among the ten employed a three-item measure, also developed by the authors, that examined adolescents’ exposure to interparental violence to investigate its impact on DV perpetration (Ontiveros et al., 2020). In the final remaining study (Morris et al., 2015), interparental violence was assessed using a combination of four items from the Children’s Perception of Interparental Conflict Scale (Grych et al., 1992) and the Conflict Tactics Scale (Straus, 1979). Finally, the same study (Morris et al., 2015) examined *harsh discipline* (i.e., experiencing yelling, slapping, or hitting from their parents) as a predictor of DV perpetration among adolescents using a three-item measure (Ge et al., 1994).

### Measurement of DV Perpetration

Overall, *n* = 6 (43%) studies (Foshee et al., 2015, 2016; Gottfredson et al., 2022; Morris et al., 2015; Reyes & Foshee, 2013) used the Safe Dates Abuse measure to assess physical DV and/or psychological DV perpetration (Foshee, 1996). Both the Safe Dates physical and psychological perpetration measures were developed for the Safe Dates program, which is a school-based teen DV prevention program for middle and high school students (Foshee et al., 2015, 2016; Gottfredson et al., 2022; Morris et al., 2015; Reyes & Foshee, 2013).

In addition, *n* = 5 (36%) studies employed the Conflict in Adolescent Dating Relationships Inventory (CADRI) (Davis et al., 2019; Latzman et al., 2015; Moretti et al., 2014; Ontiveros et al., 2020; Temple, Shorey, Fite, et al., 2013; Temple, Shorey, Tortolero, et al., 2013; Wolfe et al., 2001). Of these five studies, two studies only assessed physical DV perpetration by utilizing the four-item physical DV perpetration subscale from CADRI (Ontiveros et al., 2020; Temple, Shorey, Fite, et al., 2013) and the other three studies assessed all types of DV perpetration measured by the CADRI (i.e., physical DV, psychological DV, sexual DV, threatening, verbal, and relational (Davis et al., 2019; Latzman et al., 2015; Temple, Shorey, Tortolero, et al., 2013).

Moreover, two of the included studies assessed physical, psychological (A. Mennicke et al., 2021), and sexual DV perpetration (A. M. Mennicke et al., 2022) using the relevant measure items related to the mentioned DV types from the National IPV and Sexual Violence Survey (NISVS) (Black et al., 2011). The NISVS survey is an ongoing national survey conducted by the CDC to investigate the incidence of IPV, stalking, or sexual violence among adult men and women; however, these two particular studies adapted the items from this survey to be appropriate for high school students (A. Mennicke et al., 2021; A. M. Mennicke et al., 2022). Finally, the remaining study employed a modified version of the Conflict Tactics Scale to measure physical DV perpetration among female adolescents (Moretti et al., 2014).

### Impact of HD on DV Perpetration

#### Family Conflict

The results of the impact of HD on DV perpetration among the included studies are presented in (Supplemental Material Appendix B: Garrard Matrix of Included Articles (*N* = 14)). Four (29%) of the retained studies examined the association between family conflict (i.e., yelling, getting angry, or fighting among family members) from the HD domain and several forms of DV perpetration (i.e., physical, relational, verbal, sexual, threatening, and psychological DV) (Davis et al., 2019; Foshee et al., 2015, 2016; Gottfredson et al., 2022). Out of the four studies, three studies found a significant association between experiencing family conflict and engaging in DV perpetration among adolescents (Foshee et al., 2015, 2016; Gottfredson et al., 2022). For example, Foshee et al. (2016) found that family conflict was one of the significant risk factors that had a homogenous main effect on all three outcomes assessed in the study, including physical DV perpetration, sexual harassment, and bullying.

Similarly, another study conducted by Foshee et al. (2015) reported that family conflict was a significant risk factor that had a homogenous main effect on outcomes, including physical DV perpetration and peer violence among adolescent girls. Additionally, family conflict also had a significant homogeneous main effect regarding physical DV perpetration and peer violence among adolescent boys (Foshee et al., 2015). Finally, the longitudinal study conducted in North Carolina, which assessed the latent trajectory patterns of DV perpetration, non-violent deviance, peer violence perpetration, and alcohol usage during adolescence as predictors of adult convictions, reported a significant association between increased family conflict and adolescents perpetrating physical and psychological DV perpetration (Gottfredson et al., 2022).

#### Witnessing Parental IPV

Among the ten studies that evaluated witnessing IPV as one of the predictors of DV perpetration among adolescents, *n* = 8 (80%) studies reported significant associations between exposure to parental IPV and various forms of DV perpetration among adolescents. For example, one cross-sectional study out of the eight found a significant association between exposure to parental IPV and perpetrating relational abuse both in baseline and during the 5-month follow-up among middle school adolescents (Latzman et al., 2015).

Five studies that examined the relationship between physical DV perpetration and witnessing parental IPV among adolescents all reported a significant relationship (Moretti et al., 2014; Morris et al., 2015; Ontiveros et al., 2020; Reyes et al., 2015; Temple, Shorey, Fite, et al., 2013). Two studies out of the eight reported a significant relationship between witnessing parental IPV and physical and psychological DV perpetration among adolescents (A. Mennicke et al., 2021; Temple, Shorey, Tortolero, et al., 2013).

#### Harsh Discipline

One study that assessed parental harsh discipline (i.e., experiencing yelling, spanking, or slapping from parents) reported a significant correlation with physical DV perpetration (Morris et al., 2015).

#### Study Rigor Indices

The study rigor indices are presented in Table 2. Overall, all included studies reported their study objectives, study design, how the sample was selected, utilized valid measures, and reported statistical significance. However, these 14 studies did not report a statistical power estimate of the sample nor survey response rates. In addition, *n* = 10 (71%) studies reported CI; however, only one out of the included studies reported sample generalizability. Most studies did not mention selection bias as a limitation *n* = 12 (86%). The quality score of the studies ranged from five to seven out of ten points, with *n* = 4 (29%) studies with a score of five, *n* = 8 (57%) studies with a score of six, and *n* = 2 (14%) studies with a quality score of seven. Studies with higher quality scores indicate moderate rigor.

## Discussion

To our knowledge, this is the first scoping review to describe existing literature regarding the impact of HD on DV perpetration among adolescents aged 10 to 17 years in the United States. We reviewed 14 studies and found that most (*n* = 11) of the included studies that were conducted from 2013 to August 2023 found a significant association between HD and DV perpetration among adolescents in the United States (A. Mennicke et al., 2021; Foshee et al., 2015, 2016; Gottfredson et al., 2022; Reyes et al., 2015; Latzman et al., 2015; Moretti et al., 2014; Morris et al., 2015; Ontiveros et al., 2020; Temple, Shorey, Fite, et al., 2013; Temple, Shorey, Tortolero, et al., 2013). Findings from this review would help inform primary and secondary DV perpetration prevention interventions for adolescents and youth.

In this scoping review, the three most common types of HD that were assessed as an independent predictor of adolescent DV perpetration were family conflict, witnessing parental IPV or interparental violence, and harsh discipline. This exploration of the HD domain across these studies indicated that HD can be measured as an independent construct rather than only a sub-construct of ACEs. Often, HD is embedded under the broader paradigm of ACEs and is measured compositely, with individuals receiving an overall score based on their ACE exposures rather than domain-specific scores (McLennan et al., 2020; Mersky et al., 2017). However, it should be noted that these studies did not specifically define the items as a part of the HD domain but measured them as individual constructs using various instrument(s) that have several similarities with the HD domain that are usually assessed under ACEs. It is evident that HD is an integral component of ACEs; however, it can be challenging to define the HD construct due to the lack of a clear definition in the existing literature and the nuances of how youth internalize or externalize their traumatic experiences. Furthermore, as HD is a latent construct, it is challenging to encapsulate every aspect within a single definition. Thus, our HD definition is grounded in existing literature evidence. It should also be noted that although the studies only examined family conflict, witnessing IPV, and harsh discipline as individual constructs, most of these predictors used similarly phrased items as the corresponding HD items included across many ACEs instrument(s) (BRFSS, 2021; NSCH, 2021; PEARLS, 2019). Furthermore, one of the included studies examined overall ACEs and exposure to interparental violence as separate predictors of DV perpetration, which shows the complexity of these constructs and how more psychometric research is needed to set clear definitions and sub-categories of these latent constructs (Ontiveros et al., 2020).

The included studies did not explore other aspects of the HD domain (i.e., death in the family, household member(s) with mental and/or substance use disorder, divorce/separation, family criminal history, and household member(s) living with a disability). This may be because there is more precedence regarding the association between adolescents witnessing IPV or family conflict in their household and adapting those learned violent behaviors and applying them in their romantic relationships (A. Mennicke et al., 2021; Foshee et al., 2015, 2016; Gottfredson et al., 2022; Reyes et al., 2015; Latzman et al., 2015; Moretti et al., 2014; Morris et al., 2015; Ontiveros et al., 2020; Temple, Shorey, Fite, et al., 2013; Temple, Shorey, Tortolero, et al., 2013). Thus, it is imperative for future research to explore other exposures within the HD domain and examine their impact on DV and other health-related outcomes, particularly among adolescents, to have a broader understanding of the overall impact of different types of HD, as the literature remains scant.

Regarding DV perpetration, most of the included studies examined physical and psychological DV perpetration among adolescents (Foshee et al., 2015, 2016; Gottfredson et al., 2022; A. Mennicke et al., 2021; Moretti et al., 2014; Morris et al., 2015; Ontiveros et al., 2020; Reyes & Foshee, 2013; Reyes et al., 2015; Temple, Shorey, Fite, et al., 2013; Temple, Shorey, Tortolero, et al., 2013). This may be because adolescents tend to witness these types of violent behaviors in their households more than witnessing sexual DV, stalking, or digital DV behaviors among their household members. Furthermore, compared to the various instruments that the studies used to measure HD, the included studies primarily employed the Safe Dates Abuse measures and the CADRI to examine several forms of DV perpetration as the outcome(s). This demonstrates that there are already existing reliable and valid instruments to measure DV behaviors, especially for adolescents. However, one of the included studies discussed the significance of how DV is measured, whether as a continuous variable with a sum score or as a categorical variable (Ontiveros et al., 2020). This is of interest because Ontiveros et al. (2020) found that when DV perpetration was scored in a summative manner, female adolescents were found to perpetrate DV at a higher level to their partners compared to males; yet, when DV was measured categorically, the amplification of these scores reduced drastically, and females were no longer with the higher DV perpetration scores. Thus, it is important to consider a pragmatic method and justifications from the literature to select an appropriate measurement approach to minimize the chances of reporting misleading results.

There are other methodological concerns regarding some of the included studies, which limited the generalizability of their findings to all adolescents in the United States. For example, although most of the studies included a moderate to large sample size, their findings had limited generalizability to the particular regions (i.e., North Carolina, Kentucky, Rio Grande Valley, Houston, youth residing in rural counties, or high-risk metropolitan areas) and were not representative of all adolescents (Davis et al., 2019; Foshee et al., 2015; Gottfredson et al., 2022; Latzman et al., 2015; A. Mennicke et al., 2021; Ontiveros et al., 2020; Reyes et al., 2015; Temple, Shorey, Fite, et al., 2013; Temple, Shorey, Tortolero, et al., 2013). Another study utilized a convenience sampling approach to recruit adolescents exposed to parental IPV from low socioeconomic status (SES), which is not representative of adolescents who belong to higher SES also exposed to parental IPV (Foshee et al., 2016). These findings indicate that while the studies examined the impact of HD in DV perpetration in high-risk populations across various regions with low SES backgrounds, the generalizability of the findings is limited to adolescents with these contextual factors and may not extend to all adolescents in the United States.

The relationship between HD and DV perpetration is multifaceted, as many factors have been shown to contribute to this association among adolescents (Davis et al., 2019; A. Mennicke et al., 2021; A. M. Mennicke et al., 2022; Moretti et al., 2014; Temple, Shorey, Tortolero, et al., 2013). Around 29% (4/14) of the included studies assessed several moderators and mediators regarding the relationship between HD and DV perpetration, including social support, parental monitoring, school belonging, DV acceptance, rape myths, sensitivity to rejection, and acceptability of female and male violence; 80% of these studies reported significant associations between HD and DV perpetration by analyzing the aforementioned variables as moderators or mediators (Davis et al., 2019; A. Mennicke et al., 2021; A. M. Mennicke et al., 2022; Moretti et al., 2014; Temple, Shorey, Tortolero, et al., 2013) (Supplemental Material Appendix B: Garrard Matrix of Included Articles (*N* = 14)). Previous research has also indicated family/household chaos to be a significant contributor to risky behaviors among adolescents (Chatterjee et al., 2015; Delker et al., 2020). Therefore, future research should explore these protective and risk factors among this vulnerable population to inform early intervention efforts and trauma-informed care to prevent and reduce the burden of HD-related outcomes such as DV perpetration.

### Strengths and Limitations

Previous scoping or systematic reviews have primarily focused on investigating the relationship between the overall impact of ACEs on DV perpetration in various contexts (Corey et al., 2022; Hughes et al., 2017; Malhi et al., 2020; Navarro et al., 2022). However, there is a gap in the previous reviews, as they have not specifically explored the connection between a specific ACE domain (i.e., HD) and DV perpetration, particularly among adolescents. Thus, this scoping review attempted to highlight the need to understand how the HD domain affects DV perpetration behaviors among adolescents in the United States, as this association has been understudied, particularly among the United States adolescent population. This review emphasized the need for measuring other exposures to HD to enable a broader understanding of the impact of HD on DV behaviors among adolescents.

Despite the study’s contribution to the literature, this study has several limitations. Our study findings may not be generalizable to adolescents outside of the United States as sociodemographic factors, social determinants of health, and societal and cultural issues may differentially influence the association between HD and DV perpetration in other settings. Regarding study designs, this scoping review did not include qualitative and interventional studies (i.e., assessing the impact of an intervention regarding the relationship between HD and DV perpetration). These study designs were excluded because the relationship between HD and DV is complex, and we only included quantitative studies to compare the results across the included studies. However, excluding qualitative or interventional studies from the scoping review may limit the broader understanding of this relationship. Thus, future studies may consider conducting qualitative scoping reviews and reviews on the effect of interventions on this topic, as it will provide insightful information concerning adolescents’ experiences related to DV and their association with HD. Another limitation is that despite using three primary databases for the literature search, there is a possibility of not including studies from additional databases, gray literature, dissertations, conference proceedings, government reports, and case studies, which may provide additional information related to this issue. Moreover, based on this scoping review’s eligibility criteria, it only included studies published in English, which may exclude essential studies published in other languages.

## Conclusion

This scoping review accumulated and summarized the current literature evidence regarding the impact of HD on DV perpetration among adolescents in the United States. Most of the included studies found a significant association between HD and DV perpetration among adolescents; however, the review also identified gaps particularly related to measurement issues regarding HD and the need for additional investigations regarding the HD construct to understand its overall impact on DV and other health-related outcomes. Future research addressing these issues may help establish valid and reliable psychometric measures, promote early interventions, and develop preventative policies to help foster resilience among this vulnerable population locally and globally. The critical findings and the implications for practice, policy, and research are presented in Tables 3 and 4.

**Table 3.** Critical Findings.

Critical Findings
• Seventy-nine percent of the included studies found a significant association between HD and various types of DV perpetration (i.e., physical, psychological, sexual, relational, verbal, and threatening). • The three most common types of HD that were assessed as an independent predictor of DV perpetration were family conflict, witnessing parental IPV, and harsh discipline. • Other aspects of HD (i.e., death in the family, household member(s) with mental and/or substance use disorder, divorce/separation, family criminal history, and household member(s) living with a disability) were not examined. • The included studies did not use an HD measure; instead, they utilized various instruments (with similarly phrased items) specific to the individual constructs of HD they assessed. • The majority of the studies used the CADRI or Safe Dates Abuse measure to assess DV perpetration. • The relationship between HD and DV perpetration is multifaceted, as there are underlying factors that may contribute to this association. For example, 29% of the studies evaluated the role of several moderators and mediators (i.e., protective factors, rape myths, DV acceptance, and more); 80% of those studies reported these factors to have a significant role regarding the relation between HD and DV perpetration among adolescents.

*Note.* CADRI = Conflict in Adolescent Dating Relationships Inventory; DV = dating violence; HD = household dysfunction; IPV = intimate partner violence.

**Table 4.** Implications for Practice, Policy, and Research.

Implications for Practice, Policy, and Research
• There is limited empirical evidence regarding the impact of HD on DV perpetration behaviors among adolescents. Thus, future research is warranted regarding examining additional HD-related elements to better understand its cumulative effect on these behaviors. • There is a need for improved domain-specific age-appropriate HD measures for adolescents. Future research is needed to establish valid and reliable psychometric HD measures to understand its overall impact on DV behaviors and other health-related outcomes. • HD- and DV-related early interventions and the development of preventative policies should be undertaken to mitigate immediate and long-term consequences related to these issues and foster resilience among adolescents.

*Note.* DV = dating violence; HD = household dysfunction.

## Supplemental Material

sj-docx-1-tva-10.1177_15248380241277267 – Supplemental material for The Impact of Household Dysfunction on Dating Violence Perpetration Among Adolescents in the United States: A Scoping Review Supplemental material, sj-docx-1-tva-10.1177_15248380241277267 for The Impact of Household Dysfunction on Dating Violence Perpetration Among Adolescents in the United States: A Scoping Review by Sumaita Choudhury, Melissa F. Peskin, Timothy J. Walker, Emily T. Hébert, Nivedhitha Parthasarathy, Kaitlyn L. Zajack-Garcia, Lea Sacca and Christine M. Markham in Trauma, Violence, & Abuse

## References

[bibr1-15248380241277267] Afifi T. O. Salmon S. Garcés I. Struck S. Fortier J. Taillieu T. Stewart-Tufescu A. Asmundson G. J. G. Sareen J. MacMillan H. L. (2020). Confirmatory factor analysis of adverse childhood experiences (ACEs) among a community-based sample of parents and adolescents. BMC Pediatrics, 20(1), 178. 10.1186/s12887-020-02063-3 32316954 PMC7171813

[bibr2-15248380241277267] Arksey H. O’Malley L. (2005). Scoping studies: Towards a methodological framework. International Journal of Social Research Methodology, 8(1), 19–32. 10.1080/1364557032000119616

[bibr3-15248380241277267] Basile K. C. Black M. C. Simon T. R. Arias I. Brener N. D. Saltzman L. E. (2006). The association between self-reported lifetime history of forced sexual intercourse and recent health-risk behaviors: Findings from the 2003 National Youth Risk Behavior Survey. Journal of Adolescent Health, 39(5), 752.e1–752.e7. 10.1016/j.jadohealth.2006.06.001 17046513

[bibr4-15248380241277267] Black M. C. Basile K. C. Breiding M. J. Smith S. G. Walters M. L. Merrick M. T. Chen J. Stevens M. R. (2011). The National Intimate Partner and Sexual Violence Survey (NISVS): 2010 Summary Report. National Center for Injury Prevention and Control, Centers for Disease Control and Prevention. https://www.cdc.gov/violenceprevention/pdf/nisvs_report2010-a.pdf

[bibr5-15248380241277267] Bloom B. L. (1985). A factor analysis of self-report measures of family functioning. Family Process, 24(2), 225–239. 10.1111/j.1545-5300.1985.00225.x 4018243

[bibr6-15248380241277267] Boyce S. C. Deardorff J. McGlone L. Minnis A. M. (2023). Multi-level protective and risk factors longitudinally associated with dating violence perpetration among non-urban Mexican-American Adolescents. Adolescents, 3(1), 72–81. 10.3390/adolescents3010005 38405681 PMC10888527

[bibr7-15248380241277267] Bussemakers C. Kraaykamp G. Schoon I. Tolsma J. (2022). Household dysfunction and child development: Do financial resources matter? Advances in Life Course Research, 51, 100447. 10.1016/j.alcr.2021.100447 36652310

[bibr8-15248380241277267] BRFSS. (2021). BRFSS Adverse Childhood Experience (ACE) Module. 1.

[bibr9-15248380241277267] CDC. (2022, May 31). Fast facts: Preventing teen dating violence |Violence Prevention|Injury Center|CDC. https://www.cdc.gov/violenceprevention/intimatepartnerviolence/teendatingviolence/fastfact.html

[bibr10-15248380241277267] CDC. (2023, May 2). Youth risk behavior surveillance system (YRBSS). https://www.cdc.gov/healthyyouth/data/yrbs/index.htm

[bibr11-15248380241277267] Center for Evidence-Based Management. (2014). Critical appraisal checklist. https://cebma.org/resources-and-tools/

[bibr12-15248380241277267] Chatterjee A. Gillman M. W. Wong M. D. (2015). Chaos, Hubbub, and order scale and health risk behaviors in adolescents in Los Angeles. The Journal of Pediatrics, 167(6), 1415–1421. 10.1016/j.jpeds.2015.08.043 26394824 PMC6023620

[bibr13-15248380241277267] Clayton H. B. (2023). Dating violence, sexual violence, and bullying victimization among high school students—Youth risk behavior survey, United States, 2021. MMWR Supplements, 72, 66–74. 10.15585/mmwr.su7201a8 37104527 PMC10156153

[bibr14-15248380241277267] Collins W. A. (2003). More than myth: The developmental significance of romantic relationships during adolescence. Journal of Research on Adolescence, 13(1), 1–24. 10.1111/1532-7795.1301001

[bibr15-15248380241277267] Corey J. Duggan M. Travers Á . (2022). Risk and protective factors for intimate partner violence against bisexual victims: A systematic scoping review. Trauma, Violence, & Abuse, 24(4), 2130–2142. 10.1177/15248380221084749 PMC10486155 35435063

[bibr16-15248380241277267] Davis J. P. Ports K. A. Basile K. C. Espelage D. L. David-Ferdon C. F. (2019). Understanding the buffering effects of protective factors on the relationship between adverse childhood experiences and teen dating violence perpetration. Journal of Youth and Adolescence, 48(12), 2343–2359. 10.1007/s10964-019-01028-9 31041619 PMC6821577

[bibr17-15248380241277267] Delker E. East P. Blanco E. Wu V. Encina P. Lozoff B. Delva J. Gahagan S. (2020). Associations among household chaos, school belonging and risk behaviors in adolescents. The Journal of Primary Prevention, 41(4), 383–396. 10.1007/s10935-020-00592-2 32623561 PMC7942815

[bibr18-15248380241277267] Duke N. N. Pettingell S. L. McMorris B. J. Borowsky I. W. (2010). Adolescent violence perpetration: Associations with multiple types of adverse childhood experiences. Pediatrics, 125(4), e778–e786. 10.1542/peds.2009-0597 20231180

[bibr19-15248380241277267] Felitti V. J. Anda R. F. Nordenberg D. Williamson D. F. Spitz A. M. Edwards V. Koss M. P. Marks J. S. (2019). REPRINT OF: Relationship of childhood abuse and household dysfunction to many of the leading causes of death in adults: The Adverse Childhood Experiences (ACE) Study. American Journal of Preventive Medicine, 56(6), 774–786. 10.1016/j.amepre.2019.04 31104722

[bibr20-15248380241277267] Fonseka R. W. Minnis A. M. Gomez A. M. (2015). Impact of adverse childhood experiences on intimate partner violence perpetration among Sri Lankan men. PLoS One, 10(8), e0136321. 10.1371/journal.pone.0136321 PMC4546656 26295577

[bibr21-15248380241277267] Foshee V. A. (1996). Gender differences in adolescent dating abuse prevalence, types and injuries. Health Education Research, 11(3), 275–286. 10.1093/her/11.3.275-a

[bibr22-15248380241277267] Foshee V. A. McNaughton Reyes L. Tharp A. T. Chang L.-Y. Ennett S. T. Simon T. R. Latzman N. E. Suchindran C. (2015). Shared longitudinal predictors of physical peer and dating violence. The Journal of Adolescent Health: Official Publication of the Society for Adolescent Medicine, 56(1), 106–112. 10.1016/j.jadohealth.2014.08.003 25287983 PMC10903635

[bibr23-15248380241277267] Foshee V. A. McNaughton Reyes H. L. Chen M. S. Ennett S. T. Basile K. C. DeGue S. Vivolo-Kantor A. M. Moracco K. E. Bowling J. M. (2016). Shared risk factors for the perpetration of physical dating violence, bullying, and sexual harassment among adolescents exposed to domestic violence. Journal of Youth and Adolescence, 45(4), 672–686. 10.1007/s10964-015-0404-z 26746242 PMC5859571

[bibr24-15248380241277267] Garrard J. (2020). Health sciences literature review made easy. Jones & Bartlett Learning.

[bibr25-15248380241277267] Ge X. Conger R. D. Lorenz F. O. Simons R. L. (1994). Parents’ stressful life events and adolescent depressed mood. Journal of Health and Social Behavior, 35(1), 28–44. 10.2307/2137333 8014428

[bibr26-15248380241277267] Gottfredson N. C. McNaughton-Reyes H. L. Wu J. (2022). Predictive associations between adolescent profiles of violent and nonviolent deviant behavior with convictions in adulthood. Journal of Interpersonal Violence, 37(13–14), NP12207–NP12237. 10.1177/0886260521997453 33682492

[bibr27-15248380241277267] Grych J. H. Seid M. Fincham F. D. (1992). Assessing marital conflict from the child’s perspective: The children’s perception of interparental conflict scale. Child Development, 63(3), 558–572. 10.2307/1131346 1600822

[bibr28-15248380241277267] Hamby S. Finkelhor D. Turner H. Kracke K. (2011). Juvenile victimization questionnaire. Crimes Against Children Research Center. https://www.unh.edu/ccrc/juvenile-victimization-questionnaire

[bibr29-15248380241277267] Haynie D. L. Farhat T. Brooks-Russell A. Wang J. Barbieri B. Iannotti R. J. (2013). Dating Violence perpetration and victimization among US Adolescents: Prevalence, patterns, and associations with health complaints and substance use. The Journal of Adolescent Health: Official Publication of the Society for Adolescent Medicine, 53(2), 194–201. 10.1016/j.jadohealth.2013.02.008 23664626 PMC3725188

[bibr30-15248380241277267] Hughes K. Bellis M. A. Hardcastle K. A. Sethi D. Butchart A. Mikton C. Jones L. Dunne M. P. (2017). The effect of multiple adverse childhood experiences on health: A systematic review and meta-analysis. The Lancet Public Health, 2(8), e356–e366. 10.1016/S2468-2667(17)30118-4 29253477

[bibr31-15248380241277267] Jouriles E. N. McDonald R. Mueller V. Grych J. H. (2012). Youth experiences of family violence and teen dating violence perpetration: Cognitive and emotional mediators. Clinical Child and Family Psychology Review, 15(1), 58–68. 10.1007/s10567-011-0102-7 22160838

[bibr32-15248380241277267] Latzman N. E. Vivolo-Kantor A. M. Holditch Niolon P. Ghazarian S. R. (2015). Predicting adolescent dating violence perpetration: Role of exposure to intimate partner violence and parenting practices. American Journal of Preventive Medicine, 49(3), 476–482. 10.1016/j.amepre.2015.06.006 26296446 PMC5839136

[bibr33-15248380241277267] Malhi N. Oliffe J. L. Bungay V. Kelly M. T. (2020). Male perpetration of adolescent dating violence: A scoping review. American Journal of Men’s Health, 14(5), 1557988320963600. 10.1177/1557988320963600 PMC7557791 33045903

[bibr34-15248380241277267] McGee R. A. Wolfe D. A. Wilson S. K. (1997). Multiple maltreatment experiences and adolescent behavior problems: Adolescents’ perspectives. Development and Psychopathology, 9(1), 131–149. 10.1017/s0954579497001107 9089128

[bibr35-15248380241277267] McLennan J. D. MacMillan H. L. Afifi T. O. (2020). Questioning the use of adverse childhood experiences (ACEs) questionnaires. Child Abuse & Neglect, 101, 104331. 10.1016/j.chiabu.2019.104331 31887655

[bibr36-15248380241277267] Mennicke A. Bush H. M. Brancato C. J. Coker A. L. (2021). Bystander intervention efficacy to reduce teen dating violence among high school youth who did and did not witness parental partner violence: A path analysis of a cluster RCT. Journal of Family Violence, 36(7), 755–771. 10.1007/s10896-021-00297-y 34776603 PMC8550687

[bibr37-15248380241277267] Mennicke A. M. Bush H. M. Brancato C. J. Coker A. L. (2022). Bystander program to reduce sexual violence by witnessing parental intimate partner violence status. American Journal of Preventive Medicine, 63(2), 262–272. 10.1016/j.amepre.2021.12.022 35279345

[bibr38-15248380241277267] Mersky J. P. Janczewski C. E. Topitzes J. (2017). Rethinking the measurement of adversity: Moving toward second-generation research on adverse childhood experiences. Child Maltreatment, 22(1), 58–68. 10.1177/1077559516679513 27920222

[bibr39-15248380241277267] Moretti M. M. Bartolo T. Craig S. Slaney K. Odgers C. (2014). Gender and the transmission of risk: A prospective study of adolescent girls exposed to maternal versus paternal interparental violence. Journal of Research on Adolescence, 24(1), 80–92. 10.1111/jora.12065

[bibr40-15248380241277267] Morris A. M. Mrug S. Windle M. (2015). From family violence to dating violence: Testing a dual pathway model. Journal of Youth and Adolescence, 44(9), 1819–1835. 10.1007/s10964-015-0328-7 26208831 PMC6679925

[bibr41-15248380241277267] Navarro R. Larrañaga E. Yubero S. Víllora B. (2022). Associations between adverse childhood experiences within the family context and in-person and online dating violence in adulthood: A scoping review. Behavioral Sciences, 12(6), 162. 10.3390/bs12060162 35735372 PMC9219904

[bibr42-15248380241277267] NSCH, U. C. (2021). National Survey of Children’s Health (NSCH). Census.Gov. https://www.census.gov/nsch

[bibr43-15248380241277267] Ontiveros G. Cantos A. Chen P.-Y. Charak R. O’Leary K. D. (2020). Is all dating violence equal? Gender and severity differences in predictors of perpetration. Behavioral Sciences, 10(7), 118. 10.3390/bs10070118 32698435 PMC7407285

[bibr44-15248380241277267] PEARLS. (2019). Pediatric ACEs and Related Life Events Screener (PEARLS). 3. 10.1007/s40653-025-00774-2 PMC13546261 42703317

[bibr45-15248380241277267] Peskin M. F. Markham C. M. Shegog R. Baumler E. R. Addy R. C. Temple J. R. Hernandez B. Cuccaro P. M. Thiel M. A. Gabay E. K. Tortolero Emery S. R. (2019). Adolescent dating violence prevention program for early adolescents: The me & you randomized controlled trial, 2014–2015. American Journal of Public Health, 109(10), 1419–1428. 10.2105/AJPH.2019.305218 31415194 PMC6727296

[bibr46-15248380241277267] Rayyan. (n.d.). Retrieved December 10, 2022, from https://rayyan.ai/reviews/539757

[bibr47-15248380241277267] Reidy D. E. Smith-Darden J. P. Cortina K. S. Kernsmith R. M. Kernsmith P. D. (2015). Masculine discrepancy stress, teen dating violence, and sexual violence perpetration among adolescent boys. Journal of Adolescent Health, 56(6), 619–624. 10.1016/j.jadohealth.2015.02.009 PMC5859556 26003576

[bibr48-15248380241277267] Reyes H. L. M. Foshee V. A. (2013). Sexual dating aggression across grades 8 through 12: timing and predictors of onset. Journal of Youth and Adolescence, 42(4), 581–595. 10.1007/s10964-012-9864-6 23180071 PMC3596483

[bibr49-15248380241277267] Reyes H. L. M. Foshee V. A. Fortson B. L. Valle L. A. Breiding M. J. Merrick M. T. (2015). Longitudinal mediators of relations between family violence and adolescent dating aggression perpetration. Journal of Marriage and the Family, 77(7), 1016–1030. 10.1111/jomf.12200 26719602 PMC4692054

[bibr50-15248380241277267] Reyes M. E. Simpson L. Sullivan T. P. Contractor A. A. Weiss N. H. (2021). Intimate partner violence and mental health outcomes among hispanic women in the United States: A scoping review. Trauma, Violence, & Abuse, 24(2), 809–827. 10.1177/15248380211043815 PMC13110112 34779327

[bibr51-15248380241277267] Rothman E. F. Xuan Z. (2014). Trends in physical dating violence victimization among U.S. high school students, 1999–2011. Journal of School Violence, 13(3), 277–290. 10.1080/15388220.2013.847377 25143760 PMC4134915

[bibr52-15248380241277267] Shorey R. C. Allan N. P. Cohen J. R. Fite P. J. Stuart G. L. Temple J. R. (2019). Testing the factor structure and measurement invariance of the conflict in adolescent dating, relationship inventory. Psychological Assessment, 31(3), 410–416. 10.1037/pas0000678 30589276 PMC6389429

[bibr53-15248380241277267] Simon V. A. Aikins J. W. Prinstein M. J. (2008). Romantic partner selection and socialization during early adolescence. Child Development, 79(6), 1676–1692. 10.1111/j.1467-8624.2008.01218.x 19037942 PMC3420070

[bibr54-15248380241277267] Simpson D. D. McBride A. A. (1992). Family, Friends, and Self (FFS) assessment scales for Mexican American youth. Hispanic Journal of Behavioral Science, 14, 327–340.

[bibr55-15248380241277267] Straus M. A. (1979). Measuring intrafamily conflict and violence: The Conflict Tactics (CT) scales. Journal of Marriage and Family, 41(1), 75–88. 10.2307/351733

[bibr56-15248380241277267] Temple J. R. Shorey R. C. Fite P. Stuart G. Le V. D. (2013). Substance use as a longitudinal predictor of the perpetration of teen dating violence. Journal of Youth and Adolescence, 42(4), 596–606. 10.1007/s10964-012-9877-1 23187699 PMC3670106

[bibr57-15248380241277267] Temple J. R. Shorey R. C. Tortolero S. R. Wolfe D. A. Stuart G. L. (2013). Importance of gender and attitudes about violence in the relationship between exposure to interparental violence and the perpetration of teen dating violence. Child Abuse & Neglect, 37(5), 343. 10.1016/j.chiabu.2013.02.001 PMC3670104 23490056

[bibr58-15248380241277267] Thornberry T. P. Lizotte A. J. Krohn M. D. Smith C. A. Porter P. K. (2003). Causes and consequences of delinquency. In Thornberry T. P. Krohn M. D. (Eds). Taking stock of delinquency (pp. 11–46). Kluwer Academic Publishers.

[bibr59-15248380241277267] Vagi K. J. Rothman E. F. Latzman N. E. Tharp A. T. Hall D. M. Breiding M. J. (2013). Beyond correlates: A review of risk and protective factors for adolescent dating violence perpetration. Journal of Youth and Adolescence, 42(4), 633–649. 10.1007/s10964-013-9907-7 23385616 PMC3697003

[bibr60-15248380241277267] Wincentak K. Connolly J. Card N. (2017). Teen dating violence: A meta-analytic review of prevalence rates. Psychology of Violence, 7, 224–241. 10.1037/a0040194

[bibr61-15248380241277267] Wolfe D. A. Scott K. Reitzel-Jaffe D. Wekerle C. Grasley C. Straatman A. L. (2001). Development and validation of the conflict in adolescent dating relationships inventory. Psychological Assessment, 13(2), 277–293. 11433803

[bibr62-15248380241277267] Wolfe D. A. Scott K. L. Crooks C. V. (2005). Abuse and violence in adolescent girls’ dating relationships. In Bell D. J. Foster S. L. Mash E. J. (Eds.), Handbook of behavioral and emotional problems in girls (pp. 381–414). Springer US.

