# Supplementary material for: The Impact of Household Dysfunction on Dating Violence Perpetration Among Adolescents in the United States: A Scoping Review
Source: Trauma Violence Abuse. 2024 Sep 19;26(3):377–88. doi: 10.1177/15248380241277267 (PMC12145481; doi:10.1177/15248380241277267)
Supplement: sj-docx-1-tva-10.1177_15248380241277267 – Supplemental material for The Impact of Household Dysfunction on Dating Violence Perpetration Among Adolescents in the United States: A Scoping Review [file sj-docx-1-tva-10.1177_15248380241277267.docx]

**Appendices**

**Appendix A: Search Strategy of all Databases**

| **Search Date** | **Search Strategy** |
| --- | --- |
| **Medline (Ovid)** | |
| August 16, 2023 | #1: Household Dysfunction  adverse childhood experiences/ or domestic violence/ or Exposure to Violence/ or divorce/ or family conflict/ or family separation/ or parental death/ or maternal death/ or ("adverse child* experienc*" or ACES or "witness* domestic violen*" or "witness intimate partner violen*" or "mother treated violen*" or divorc*).ti,kf,kw,ab. or ("famil* conflict*" or "interparental conflict*" or "inter-parental conflict*" or "famil* violen*" or "marital violen*" or "parent* separat*" or "harsh parent*" or "famil* separat*" or "household* dysfunct*" or "house-hold* dysfunct*" or "household* chaos*" or "house-hold* chaos*" or "household* challenge*" or "house-hold* challenge*").ti,kf,kw,ab. or ((famil* or parent* or mother* or father* or matern* or patern* or "household* member*" or "house-hold* member*" or caregiver* or sibling*) adj3 (incarcerat* or jail or death* or disabilit* or "mental* illness*" or "mental* disorder*" or "substance abus*" or "substance-related disorder*")).ti,kf,kw,ab. |
|  | #2: Adolescents  adolescent/ or child/ or Minors/ or (adolescen* or teen* or child* or minors* or juvenile* or kids* or youth* or "middle school*" or "high school*" or "junior high*" or "pre teen*" or preteen* or pubescen* or schoolchild* or "school-child*").ti,kf,kw,ab. |
|  | #3: Dating Violence Perpetration  Intimate Partner Violence/ or Stalking/ or Juvenile Delinquency/ or aggression/ or (stalk* or "physical aggress*" or "sexual aggress*" or "intimate partner violen*" or IPV or "juvenile delinqu*").ti,kf,kw,ab. or ((dating* or teen*) adj3 (violen* or initiat* or instigat* or perpetrat*)).ti,kf,kw,ab. |
|  | #4: #1 AND #2 AND #3  limit to (english language and yr="2013 - 2023") |
| **PsycINFO** | |
| August 16, 2023 | #1: Household Dysfunction  childhood adversity/ or domestic violence/ or exposure to violence/ or divorce/ or family conflict/ or marital conflict/ or family separation/ or parental death/ or dysfunctional family/ or parental absence/ or ("adverse child* experienc*" or ACES or "witness* domestic violen*" or "witness intimate partner violen*" or "mother treated violen*" or divorc*).ab,id,ti. or ("famil* conflict*" or "interparental conflict*" or "inter-parental conflict*" or "famil* violen*" or "marital violen*" or "parent* separat*" or "harsh parent*" or "famil* separat*" or "household* dysfunct*" or "house-hold* dysfunct*" or "household* chaos*" or "house-hold* chaos*" or "household* challenge*" or "house-hold* challenge*").ab,id,ti. or ((famil* or parent* or mother* or father* or matern* or patern* or "household* member*" or "house-hold* member*" or caregiver* or sibling*) adj3 (incarcerat* or jail or death* or disabilit* or "mental* illness*" or "mental* disorder*" or "substance abus*" or "substance-related disorder*")).ab,id,ti. |
|  | #2: Adolescents  (adolescen* or teen* or child* or minors* or juvenile* or kids* or youth* or "middle school*" or "high school*" or "junior high*" or "pre teen*" or preteen* or pubescen* or schoolchild* or "school-child*").ab,id,ti. |
|  | #3: Dating Violence Perpetration  Intimate Partner Violence/ or relational aggression/ or stalking/ or juvenile delinquency/ or predelinquent youth/ or dating violence/ or (stalk* or "physical aggress*" or "sexual aggress*" or "intimate partner violen*" or IPV or "juvenile delinqu*").ab,id,ti. or ((dating* or teen*) adj3 (violen* or initiat* or instigat* or perpetrat*)).ab,id,ti. |
|  | #4: #1 AND #2 AND #3  limit to (english language and yr="2013 - 2023") |
| **EMBASE** | |
| August 16, 2023 | #1: Household Dysfunction  'childhood adversity'/de OR 'domestic violence'/de OR 'exposure to violence'/de OR 'divorce'/de OR 'family conflict'/de OR 'family separation'/de OR 'parental death'/de OR 'maternal death'/de OR 'household dysfunction'/de OR 'dysfunctional family'/de OR 'adverse child* experienc*':ab,kw,ti OR aces:ab,kw,ti OR 'witness* domestic violen*':ab,kw,ti OR 'witness intimate partner violen*':ab,kw,ti OR 'mother treated violen*':ab,kw,ti OR divorc*:ab,kw,ti OR 'famil* conflict*':ab,kw,ti OR 'interparental conflict*':ab,kw,ti OR 'inter-parental conflict*':ab,kw,ti OR 'famil* violen*':ab,kw,ti OR 'marital violen*':ab,kw,ti OR 'parent* separat*':ab,kw,ti OR 'harsh parent*':ab,kw,ti OR 'famil* separat*':ab,kw,ti OR 'household* dysfunct*':ab,kw,ti OR 'house-hold* dysfunct*':ab,kw,ti OR 'household* chaos*':ab,kw,ti OR 'house-hold* chaos*':ab,kw,ti OR 'household* challenge*':ab,kw,ti OR 'house-hold* challenge*':ab,kw,ti OR ((famil* OR parent* OR mother* OR father* OR matern* OR patern* OR 'household* member*' OR 'house-hold* member*' OR caregiver* OR sibling*) NEAR/3 (incarcerat* OR jail OR death* OR disabilit* OR 'mental* illness*' OR 'mental* disorder*' OR 'substance abus*' OR 'substance-related disorder*')):ab,kw,ti |
|  | #2: Adolescents  'adolescent'/de OR 'child'/de OR 'minor (person)'/de OR adolescen*:ab,kw,ti OR teen*:ab,kw,ti OR child*:ab,kw,ti OR minors*:ab,kw,ti OR juvenile*:ab,kw,ti OR kids*:ab,kw,ti OR youth*:ab,kw,ti OR 'middle school*':ab,kw,ti OR 'high school*':ab,kw,ti OR 'junior high*':ab,kw,ti OR 'pre teen*':ab,kw,ti OR preteen*:ab,kw,ti OR pubescen*:ab,kw,ti OR schoolchild*:ab,kw,ti OR 'school-child*':ab,kw,ti |
|  | #3: Dating Violence Perpetration  'partner violence'/de OR 'stalking'/de OR 'juvenile delinquency'/de OR 'aggression'/de OR 'dating violence'/de OR stalk*:ab,kw,ti OR 'physical aggress*':ab,kw,ti OR 'sexual aggress*':ab,kw,ti OR 'intimate partner violen*':ab,kw,ti OR ipv:ab,kw,ti OR 'juvenile delinqu*':ab,kw,ti OR ((dating* OR teen*) NEAR/3 (violen* OR initiat* OR instigat* OR perpetrat*)):ab,kw,ti |
|  | #4: #1 AND #2 AND #3  Limit to (2013:py OR 2014:py OR 2015:py OR 2016:py OR 2017:py OR 2018:py OR 2019:py OR 2020:py OR 2021:py OR 2022:py OR 2023:py) AND [article]/lim AND [english]/lim |

**Appendix B: Garrard Matrix of Included Articles (*N*=14)**

| **Study #** | **Study Author(s)/**  **Year** | **Study Aim(s)** | **Study Design** | **Study Sample** | **Type(s) of HD Measured and Instrument(s)** | **Type(s) of DV perpetration Analyzed and Instrument(s)** | **Key Finding(s)**  **Association Between HD and DV Perpetration** |
| --- | --- | --- | --- | --- | --- | --- | --- |
| 1 | Davis et al., 2019 | Explored the trajectories from exposure witnessing community violence and family conflict, to DV perpetration among adolescents. The study also aimed to identify the protective factors that alleviated the association between ACEs and DV perpetration in the future. | Longitudinal | Sample size = 1,611  Mean age = 12.77 years  Adolescents from four middle schools who transitioned to high school from 2008 – 2013.  This study includes a subset of the sample population who reported being in a romantic relationship during high school. | Family conflict (i.e., three items assessed yelling, arguing, and losing temper among household members).  **Instrument:**  Family Conflict and Hostility Scale (Thornberry et al., 2003). | Teen DV perpetration (i.e., relational, verbal, physical, threatening, and sexual).  **Instrument:**  Conflict in Adolescent Dating Relationships Inventory (CADRI) (Wolfe et al., 2001). | Adolescents in decreasing family conflict/increasing community violence class with higher social support reported lower odds (AOR=0.57; 95% CI: 0.34, 0.98) of high DV perpetration and for physical/verbal DV perpetration (AOR=0.67; 95% CI: 0.55, 0.82).  Adolescents under the stable high family conflict/high community violence class with higher school belonging reported lower odds (AOR=0.77; 95% CI: 0.58, 0.94) of transitioning into physical/verbal DV perpetration class.  Adolescents under the low family conflict/low community violence class with higher social support reported lower risk (AOR=0.67; 95% CI: 0.48, 0.84) of transitioning into high DV perpetration class and decreased the odds (AOR=0.85; 95% CI: 0.76, 0.95) of transitioning into physical/verbal DV perpetration class. |
| 2 | Foshee et al., 2016 | Examined the shared risk factors of DV perpetration, sexual harassment, and bullying among adolescents who witnessed intimate partner violence (IPV) on their mothers in North Carolina. | Cross-sectional  Note: The baseline data from a randomized controlled trial dating a violence prevention program was used. | Sample size = 409 families (mother and adolescent dyads).  Adolescents' mean age = 13.6 years. | Family conflict (i.e., four items measured how often adolescents’ family members said bad things to each other, hit, got angry, or yelled at each other in the last three months).  **Instrument:**  Family Conflict (Simpson & McBride, 1992). | Physical DV perpetration (i.e., items assessed slapping, pushing/shoving, or assaulting dating partners).  **Instrument:**  Safe Dates Physical Dating Abuse Scale (Foshee, 1996). | Results indicated that family conflict (AOR = 1.60; 95% CI: 1.24, 2.08; p<0.05) was one of the significant risk factors that had a homogenous main effect (i.e., the impact of the risk factor is the same across the three outcomes) on all three outcomes, including physical DV perpetration, bullying, and sexual harassment. |
| 3 | Foshee et al., 2015 | Investigated the shared risk factors of DV perpetration and peer violence among adolescents from 8^th^ through 10^th^ grade in North Carolina. | Longitudinal | Sample size = 4,277  (adolescents who completed the questionnaire in both waves).  Mean age = not reported | Family conflict (i.e., three items assessed how often the family fought in the last three months).  **Instrument:**  Family Conflict (Bloom, 1985). | Physical DV perpetration (i.e., items assessed slapping, pushing/shoving, or assaulting dating partners).  **Instrument:**  Safe Dates Physical Dating Abuse Scale (Foshee, 1996). | Results indicated that family conflict (AOR=1.18; 95% CI: 1.10, 1.22; p<0.05) among adolescent girls and adolescent boys (AOR=1.21; 95% CI: 1.11, 1.31; p<0.05) were found to have significant homogenous main effects on both outcomes, including physical DV perpetration and peer violence. |
| 4 | Gottfredson et al., 2022 | Assessed the latent trajectory patterns of DV perpetration, non-violent deviance, peer violence perpetration, and alcohol usage and whether these factors during adolescence predicted convictions during adulthood in North Carolina. | Longitudinal  (Data collected in four waves during high school).  (Data was also collected in 2019 when participants were adults and had conviction records – but these findings are not included here based on this review’s eligibility criteria). | Sample size (adolescents) = 3,979  Mean age for adolescents (not reported, however, the adolescents were all high school students). | Family conflict (i.e., three items assessed how often the family fought in the last three months).  **Instrument:**  Family Conflict (Bloom, 1985). | Psychological DV perpetration (i.e., four items assessed saying hurtful things, insulting, isolating, and emotional dominance toward dating partners).  Physical DV perpetration (i.e., 11 items assessed slapping, hitting, beating, or pushing dating partners).  **Instrument:**  Safe Dates Physical Dating Abuse and Psychological DV Perpetration Scale (Foshee, 1996). | The bivariate results showed that DV perpetrators' class (class 2 - physical and psychological DV perpetration) reported higher levels of family conflict (Mean=1.59; Standard deviation=1.46; p<0.05). |
| 5 | Latzman et al., 2015 | Examined the association between exposure to IPV, parenting practices, and several types of DV perpetration among adolescents. | Cross-sectional  Dating Matters Middle School Intervention - The study only used survey data from baseline and five-month follow-up (both pre-interventional time frames and the longitudinal data for the intervention were currently being collected). | Sample size = 698  Mean age = not reported (6^th^ and 7^th^ graders) | Exposure to IPV (two items assessed whether adolescents witnessed or heard domestic violence between their parents or parent’s dating partner, such as beating, yelling, or hitting).  **Instrument:**  Juvenile Victimization Questionnaire (Hamby et al., 2011). | Adolescent dating violence perpetration (ADV) (i.e., threatening, verbal, emotional, relational, physical, and sexual abuse).  **Instrument:**  Conflict in Adolescent Dating Relationships Inventory (CADRI) (Wolfe et al., 2001). | Adolescents who indicated that they were in a relationship reported a significant association between exposure to IPV and perpetrating relational abuse both in baseline (β = 0.22; p<0.05) and even more likely during the five-month follow-up. |
| 6 | Mennicke et al., 2021 | Investigated whether the Green Dot bystander intervention program decreased psychological and physical DV perpetration among adolescents who witnessed or did not witness IPV among their parents in Kentucky. | Longitudinal  Phase 0 = Baseline (when no intervention was implemented)  Findings from Phases 1 and 2 are not included due to this review’s eligibility criteria. | Sample size = 7,885 (intervention group) and 7,978 (control group) in Phase 0.  Mean age = not reported  However, the age range was reported to be 14-18 years old. | Witnessed parental IPV (i.e., one item assessed whether adolescents witnessed hitting, slapping, or punching between their parents).  **Instrument:**  A one-item measure that assessed the number of times adolescents had witnessed parents hit each other (Mennicke et al., 2021). | Psychological DV perpetration (i.e., three items assessed psychological DV perpetration such as threatening to hit or controlling behaviors).  Physical DV perpetration (one item assessed hitting, slapping, or physically hurting dating partner(s).  **Instrument:**  National Intimate Partner and Sexual Violence Survey (Black et al., 2011). | Results indicated that during baseline (Phase 0), there was a significant association between psychological DV perpetration and adolescents who did not witness IPV (Mean=0.41; Standard deviation=1.44; p<.001). A significant association was also found between physical DV perpetration and adolescents who did not witness IPV (Mean=0.11; Standard deviation=0.60; p<.001).  Mediation Results:  Rape myths significantly mediated the relationship between witnessing IPV and physical DV perpetration. DV acceptance also significantly mediated the relationship between witnessing IPV and psychological and physical DV perpetration during baseline. |
| 7 | Mennicke et al., 2022 | Assessed the impact of the Green Dot bystander intervention program regarding the decrease of sexual violence among high school adolescents who either did not witness or witnessed IPV among their parents in Kentucky. | Cross-sectional  Phase 0 = Baseline (when no intervention was implemented)  Findings from Phases 1 and 2 are not included due to this review’s eligibility criteria. | Sample size = 15,863 in Phase 0.  Mean age = not reported  However, the age range was reported to be 14-18 years old. | Witnessed parental IPV (i.e., one item assessed whether adolescents witnessed hitting, slapping, or punching between their parents).  **Instrument:**  A one-item measure that assessed the number of times adolescents had witnessed parents hit each other (Mennicke et al., 2022). | Sexual DV perpetration (i.e., three items assessed sexual assault, three items assessed sexual harassment, and three items measured stalking toward dating partners).  **Instrument:**  National Intimate Partner and Sexual Violence Survey (Black et al., 2011). | Results indicated that during baseline, there was an association between sexual assault, sexual harassment, and stalking perpetration among adolescents who witnessed or did not witness parental IPV within each control and intervention groups. However, none of the associations were significant.  Mediation Results:  Rape myths and DV acceptance significantly mediated the relationship between witnessing IPV and sexual assault perpetration, sexual harassment perpetration, and stalking perpetration during baseline. |
| 8 | Moretti et al., 2014 | Examined the effect of exposure to interparental violence on DV perpetration and whether sensitivity to interpersonal rejection mediated this relationship among females in romantic relationships during adolescence and early adulthood. | Longitudinal  Time 1 = adolescent females.  (Two-year and five-year follow-up findings are not included here as the mean age of the sample does not meet this review’s eligibility criteria). | Sample size (Time 1) = 139.  Mean age (Time 1) = 16.26 years. | Witnessing interparental violence between caregivers and their partners (i.e., four items assessed exposure to hitting, threatening, throwing, or pushing between parents or caregivers).  **Instrument:**  The Family Background Questionnaire (McGee et al., 1997). | Romantic partner aggression (i.e., destroyed or threatened to destroy something worthy to a dating partner, pushed or shoved a dating partner).  **Instrument:**  Conflict Tactics Scale (Straus,1979).  Conflict Tactics Scale-Revised (Straus et al., 1996). | The bivariate results showed a significant correlation between maternal IPV and Time 1 aggression towards a romantic partner (r=0.23; p<.01). However, there is no significant relationship between paternal IPV and Time 1 aggression towards a romantic partner.  Mediation Results  Sensitivity to rejection significantly mediated the relationship between exposure to maternal IPV and engaging in romantic aggression. |
| 9 | Morris et al., 2015 | Examined how exposure to interparental violence during pre-adolescence is associated with aggression, peer aggression, pro-violent beliefs, and violence towards opposite-sex peers, and how these factors are associated with DV perpetration during late adolescence. | Longitudinal  Note: The findings for waves 1 to 2 are included here. The findings from Wave 3 are not included here as the sample is aged 18 years based on this review’s eligibility criteria. | Sample size = 704 (Wave 1) and 603 (Wave 2).  Mean age = 11.8 years (Wave 1) and 13.2 years (Wave 2). | Interparental violence (four items assessed witnessing parent-to-parent violence such as pushing, showing, or hitting in the past year)  Harsh discipline (three items examined adolescents experiencing harsh discipline from their parents such as yelling, spanking, slapping, or hitting).  **Instruments:**  Children’s Perception of Interparental Conflict Scale (Grych et al.1992).  Conflict Tactics Scale (Straus,1979).  Harsh discipline (three-item scale) (Ge et al., 1994). | Early romantic aggression perpetration (i.e., 15 items assessed engaging in early romantic perpetration, including pushing, grabbing, choking, or shoving in the past 12 months).  **Instrument:**  Safe Dates Physical Perpetration Scale (Foshee et al.,1996). | The bivariate correlation findings demonstrated that there was a significant positive correlation between interparental violence in (Wave 1) and early romantic aggression perpetration in (Wave 1) (r=0.26; p<.05) and in (Wave 2) (r=0.21; p<.05).  Moreover, a significant correlation was found between harsh discipline in (Wave 1) and early romantic aggression perpetration in (Wave 1) (r=0.29; p<.05) and (Wave 2) (r=0.20; p<.05). |
| 10 | Ontiveros et al., 2020 | Investigated the relations among ACEs, exposure to interparental violence, impulsivity, emotional instability, attachment, and physical DV perpetration among adolescents in Rio Grande Valley, Texas. | Cross-sectional | Sample size = 829.  Mean age = 16 years. | Exposure to interparental violence (i.e., three items assessed witnessing mother hitting father, father hitting mother, or both parents hitting each other).  **Instrument:**  The three-item measure assessed witnessing interparental violence with Cronbach’s alpha of 0.83 (Ontiveros et al., 2020). | Physical teen DV perpetration (i.e., four items assessed physical DV perpetration).  **Instrument:**  Conflict in Adolescent Dating Relationships Inventory (CADRI) (Wolfe et al., 2001). | The binary logistic regression found a significant association between DV perpetration (that used all four CADRI items) and exposure to interparental violence among male adolescents (β =2.816; p=0.003).  Results indicated that exposure to interparental violence was significantly associated with DV perpetration in category 1 (minor/moderate violence, i.e., throwing something, or slapping a dating partner) (β =7.649; p=0.048) and category 2 (i.e., kicking, hitting, or punching) among male adolescents (β =3.065; p=0.006).  Witnessing interparental violence was not significantly associated with DV perpetration among female adolescents. |
| 11 | Reyes et al., 2013 | Assessed the time period and risk factors of sexual DV perpetration among male adolescents across grades 8-12 in North Carolina. | Longitudinal  Safe Dates Prevention Program control group data (Waves 1-6) | Sample size = 459.  Mean age = not reported (8^th^ – 12^th^ graders). | Interparental violence (i.e., number of times adolescents witnessed interparental violence).  **Instrument:**  A one-item measure that assessed the number of times adolescents had witnessed parents hit each other (Reyes et al., 2013). | Sexual dating aggression (i.e., two items assessed forced dating partner to have sex or do something sexually that their dating partner disapproved of).  Physical dating aggression (i.e., 16 items assessed physical DV perpetration towards a dating partner).  **Instrument:**  Safe Dates Physical Perpetration Scale (Foshee et al.,1996). | The study found that witnessing interparental violence was marginally associated with sexual DV perpetration onset (hazard ratio=1.46; p=.06). |
| 12 | Reyes et al., 2015 | Investigated the association between witnessing IPV and DV perpetration and experiencing violence and anger among 8^th^ and 9^th^-grade adolescents in North Carolina.  Furthermore, the authors investigated whether this relationship is mediated by normative beliefs, depression, and difficulty regulating anger. | Longitudinal  Safe Dates Prevention Program control group data  Time 1= Baseline data  (Not including findings from Time 2 and Time 3 here based on this review’s eligibility criteria). | Time 1 Sample size = 1,965.  Time 1 Mean age = Not reported  (However, 49% of the baseline participants were in 8^th^ grade). | Witnessing IPV (i.e., one item assessed the number of times adolescents witnessed IPV between their parents, such as hitting).  **Instrument:**  A one-item measure assessed how often adolescents have witnessed IPV between their parents (Reyes et al., 2015). | Physical dating aggression (i.e., ten items assessed physical DV perpetration towards a dating partner).  **Instrument:**  Safe Dates Physical Perpetration Scale (Foshee et al.,1996). | Study findings demonstrated that witnessing IPV was significantly associated with physical dating aggression during Time 1 (r=.11; p<.05).  Moreover, witnessing IPV did not demonstrate any significant indirect effect on physical dating aggression through the three mediators tested in this study from single-mediator and multi-group mediator models. |
| 13 | Temple et al., 2013 | Assessed whether factors such as exposure to interparental violence and substance use predicted physical DV perpetration among adolescents in high school in Houston, Texas | Longitudinal  (Baseline = Time 1) and (1 year follow-up = Time 2) | Sample size (Time 1) = 828  Sample size (Time 2) = 734  Mean age = 15.1 years (only reported for Time 1) | Interparental violence (measured only at Time 1) (i.e., two items measured witnessing pushing, grabbing, shoving, choking, or slamming against the walls between parents)  **Instrument:**  A two-item measure that assessed the number of times adolescents have witnessed parents hit each other (Temple et al., 2013). | Teen DV perpetration (i.e., four items assessed physical perpetration such as pushing or hitting towards a dating partner in both Time 1 and Time 2)  **Instrument:**  Conflict in Adolescent Dating Relationships Inventory (CADRI) (Wolfe et al., 2001). | Results indicated that witnessing mother-to-father violence at Time 1 significantly predicted physical DV perpetration during Time 2 (β=0.11; p<.05).  Furthermore, Caucasian adolescents witnessing mother-to-father violence also significantly predicted physical DV perpetration during Time 2 (β=24; p<.01). |
| 14 | Temple et al., 2013 | Examined the relationship between exposure to interparental violence and DV perpetration among adolescents in Houston, Texas | Cross-sectional  (Data is from wave 1 of a longitudinal study). | Sample size = 917  Mean age = 15.1 years | Interparental violence (i.e., two items measured witnessing pushing, grabbing, shoving, choking, or slamming against the walls between parents.  **Instrument:**  A two-item measure that assessed the number of times adolescents have witnessed parents hit each other (Temple et al., 2013). | Teen DV physical perpetration (i.e., four items assessed physical perpetration such as pushing or hitting towards a dating partner).  Teen DV psychological perpetration (i.e., ten items assessed psychological DV perpetration such as making fun or ridiculing dating partner(s) in front of others).  **Instrument:**  Conflict in Adolescent Dating Relationships Inventory (CADRI) (Wolfe et al., 2001). | A significant correlation was found between witnessing mother-to-father violence and physical DV perpetration among both male (r=.13; p<.01) and female adolescents (r=.14, p<.01). Physical DV was significantly correlated with father-to-mother violence only among males (r=.12, p<.05).  Witnessing mother-to-father violence (r=.17; p<.01) and father-to-mother violence (r=.15; p<.01) showed significant correlation with psychological DV perpetration among males.  Among females, a significant correlation was found between psychological DV perpetration and witnessing mother-to-father violence (r=.15; p<.01) and witnessing father-to-mother violence (r=.20; p<.001). In the multiple-group model, females, witnessing father-to-mother violence was significantly associated with higher psychological DV perpetration (β=.16; p<.001).  Mediation Results:  Acceptability of female violence fully mediated the association between mother-to-father violence and psychological and physical DV perpetration among females. Acceptability of male violence fully mediated the relationship between mother-to-father violence and physical DV perpetration among males. |
